# Editorial: Innovative strategies for the management of life threatening ventricular tachyarrhythmias—from substrate analysis to differential therapy

**DOI:** 10.3389/fcvm.2023.1264493

**Published:** 2023-08-02

**Authors:** Reinhard Höltgen, Harilaos Bogossian, Dirk Bandorski

**Affiliations:** ^1^ Klinikum Westmünsterland, St. Agnes-Hospital Bocholt Rhede, Bocholt, Germany; ^2^ School of Medicine, Witten/Herdecke University, Witten, Germany; ^3^ Faculty of Medicine, Semmelweis University, Budapest, Hungary

**Keywords:** ventricular tachyarrhythmias, sudden cadiac death, structural arrhythmogenic heart diseases, electrical storm, conventional and electroanatomical mapping

**Editorial on the Research Topic** Malignant arrhythmias and trigger mechanisms

Dear readers of this special edition, dear colleagues,

Approximately 50% of all events leading to sudden cardiac death worldwide are caused by malignant ventricular arrhythmias, thus depicting a relevant clinical problem to focus on. It was and still is of fundamental importance to grasp and comprehend the respective underlying substrates and mechanisms to develop therapeutic strategies to act against this menace and to enhance our options to avoid patients to endure the consequences of potentially lethal rhythm disturbances. It has taken immense efforts to reach the previously known extent.

Where do we come from?

The pioneers of clinical electrophysiology laid the pivotal foundations to comprehend extra- and intracardiac appearances of ventricular arrhythmias. Some of these outstanding scientists should be mentioned in this context as pars pro toto. By extensive evaluation, description and interpretation of surface ECG recordings of broad complex tachycardias by Henrick (Hein) Joan Jost Wellens (“Do not panic when confronted with a broad QRS tachycardia. Obtain a 12-lead ECG.”) (1) and Pedro Brugada (2), algorithms were developed to identify tachycardias as of ventricular origin by studying the respective surface ECG nearly at a glance. And for years, William G Stevenson and his coworkers focused on the corresponding intracardiac processes to identify and comprehend the anatomical and electrophysiological substrates of complex ventricular arrhythmias. Consecutively, detailed knowledge in the field of ventricular arrhythmias increased intensely, and new insights found entrance into the management of potential malignant ventricular rhythm disturbances.

Where are we now?

Initially “classical”, but meanwhile well known diseases were regarded as forming the vast majority of the underlying diseases. Scaring tissue related to coronary artery disease, structural myocardial alterations like cardiomyopathies or inflammations, imbalanced electrolytes, endocrine malfunctions, side effects of drug therapy and so on were quite common. And also initially, counteracting potentially underlying malfunctions and antiarrhythmic drugs formed the conventional therapeutic approaches, followed over time by cardiac active implants, specially ICDs, and ablative strategies. Basic principle was and still is, of course, to avoid ventricular malignant arrhythmias and—if they occur—terminate them in order to prevent respective patients from sudden cardiac death.

Meanwhile, expertise and knowledge in the wide field of substrates leading to malignant ventricular arrhythmias and their treatment has reached a considerable extent. Hereditary disorders, channelopathies, rare infections and the results of former radiation therapy of various kinds that may cause ventricular arrhythmias attract scientific interest. Innovative diagnostic and therapeutic options like improved MRI scan- and CT-technology, sophisticated monitoring tools, so called “wearables” for different diagnostic and therapeutic purposes, innovative EP-tools for high density substrate mapping and highly specific ablative instruments using energy of different sources to apply pristine lesions proved to be highly effective in the management of ventricular tachyarrhythmias. Moreover, innovative devices for cardiac resynchronization therapy and cardiac contraction modulation to increase reduced left ventricular function play an important part to help avoiding malignant arrhythmias. Thus, preventive strategies gain more and more importance. All these innovations require an intense and ongoing debate, recently resulting in an update of the ESC Guidelines for the management of patients with ventricular arrhythmias and the prevention of sudden cardiac death (3).

Where should we go?

Increasing knowledge in various not only medical sciences such as molecular biology, genetics, computer science and electrical engineering, moreover rapid hard- and software improvements of diagnostic and therapeutic tools enable a continuous expansion of noninvasive and invasive methods and strategies to impede sudden cardiac death. An interdisciplinary cooperation combined with a short-term scientific exchange is absolutely mandatory in order to constantly exploit the complementary possibilities.

Future topics could be the improvement of battery patency and data storage capacity of implanted devices and the development and detection of suitable automatic early warning parameters against rhythm disturbances including optimized data telemetry. A future topic of interest could be the intra- and perimyocardial tissue in order to identify more than endocardial mechanisms that potentially contribute to the incidence and maintenance of ventricular arrhythmias.

What does the current edition include?

In our issue we present state of the art-contributions of experienced authors concerning diverse clinical situations, cardiac malfunctions and diseases potentially leading to ventricular arrhythmias. Fascinating case reports of unique particularities regarding anatomy or exceptional cardiac malfunctions are included. Novel therapeutic strategies are introduced, and potentially rare comorbidities described and evaluated.

In a patient with long-term hemodialysis and a history of inferior myocardial infarction due to meanwhile chronic total occlusion of the right coronary artery monomorphic VT episodes only occurred preceded by atrial fibrillation strictly during hemodialysis, so pulmonary vein isolation turned out to be a tailored treatment of ventricular tachycardia in this unusual substrate combination.

Pulmonary hypertension exerts influence on right heart morphology and pressure conditions and thus is potentially able to create an increased ventricular arrhythmia burden. In a study presented in this issue, the arrhythmogenic profile of patients suffering from pulmonary hypertension regarding life threatening ventricular arrhythmias and their influence on prognosis and survival is examined thoroughly.

Innovative mapping tools and techniques for high density mapping allow a more detailed data acquisition. A presented study compared the clinical outcomes of two different ablation strategies. The results of a cohort of patients that underwent VT ablation based on a conventional substrate analysis were compared to those of a group with ablation based on a high density mapping with a novel tool, the HD Grid mapping catheter.

You find included an investigation of the mechanisms of ventricular fibrillation threshold (VFT) in a myocardial infarction canine model, demonstrating that VFT can be increased by rapid median nerve stimulation.

Diagnostic and therapeutic algorithms in modern ICDs are designed to detect ventricular arrhythmias precisely as such and apply suitable therapies. Sometimes complex arrhythmias meet sophisticated programmed criteria, leading to conflicts that may result in therapy withhold. You find a respective case report of a patient with short-long-short-sequences and an inhibition of antitachycardiac therapy due to a program feature dealing with VT onset.

Ablation therapy is not only an option to cure patients from (monomorphic) ventricular tachycardia. The electrical storm represents an absolutely life threatening ventricular arrhythmia. You are presented an analysis of 160 consecutive patients that underwent a ventricular substrate modulation, proving that this ablative strategy is feasible, safe and effective targeting electrical storm.

A seldom, but most challenging clinical situation consists in the occurrence of electrical storm in patients with Brugada syndrome. Treatment procedures are not heterogeneous. Medical therapy (prophylactic as well as emergency drug applications), ICD-implantation and ablation procedures are under investigation and were compiled in an article regarding the management of this particular combination.

Meanwhile, Purkinje fibers could be recognized as potential origin of initiation and maintenance of ventricular fibrillation, specifically in patients without endocardiac substrates. Innovative high density mapping tools allow to identify triggers precisely, so that ablative purkinje de-networking could be applied, turning out to be safe, feasible and effective for arrhythmia suppression.

We hope you enjoy reading our special edition, assess it as interesting and inspiring, and we wish that you gain detailed information and additional knowledge concerning the topics covered in the individual articles.

Kindest regards

Reinhard Höltgen, Harilaos Bogossian and Dirk Bandorski
